# Antiviral RNA Interference Activity in Cells of the Predatory Mosquito, *Toxorhynchites amboinensis*

**DOI:** 10.3390/v10120694

**Published:** 2018-12-06

**Authors:** Claire L. Donald, Margus Varjak, Eric Roberto Guimarães Rocha Aguiar, João T. Marques, Vattipally B. Sreenu, Esther Schnettler, Alain Kohl

**Affiliations:** 1 MRC-University of Glasgow Centre for Virus Research, Glasgow, Scotland G61 1QH, UK; Margus.Varjak@glasgow.ac.uk (M.V.); Sreenu.Vattipally@glasgow.ac.uk (V.B.S.); schnettler@bnitm.de (E.S.); 2 Departamento de Bioquímica e Imunologia, Instituto de Ciências Biológicas, Universidade Federal de Minas Gerais, 6627-Pampulha-Belo Horizonte-MG, CEP 31270-901, Brazil; ericgdp@gmail.com (E.R.G.R.A.); jtm@ufmg.br (J.T.M.)

**Keywords:** RNA interference (RNAi), antiviral responses, *Toxorhynchites amboinensis*, alphavirus, virus discovery

## Abstract

Arthropod vectors control the replication of arboviruses through their innate antiviral immune responses. In particular, the RNA interference (RNAi) pathways are of notable significance for the control of viral infections. Although much has been done to understand the role of RNAi in vector populations, little is known about its importance in non-vector mosquito species. In this study, we investigated the presence of an RNAi response in *Toxorhynchites amboinensis*, which is a non-blood feeding species proposed as a biological control agent against pest mosquitoes. Using a derived cell line (TRA-171), we demonstrate that these mosquitoes possess a functional RNAi response that is active against a mosquito-borne alphavirus, Semliki Forest virus. As observed in vector mosquito species, small RNAs are produced that target viral sequences. The size and characteristics of these small RNAs indicate that both the siRNA and piRNA pathways are induced in response to infection. Taken together, this data suggests that *Tx. amboinensis* are able to control viral infections in a similar way to natural arbovirus vector mosquito species. Understanding their ability to manage arboviral infections will be advantageous when assessing these and similar species as biological control agents.

## 1. Introduction

*Toxorhynchites* (*Diptera: Culicidae*) mosquitoes or “elephant mosquitoes” are the largest mosquitoes on the planet, with a wingspan surpassing 12 mm for some species [1]. Unlike most mosquito species, they are autogenous and do not require a blood meal for egg production. Instead, all instars of larvae are predatory against other mosquito larvae, including those of medical relevance, such as *Aedes aegypti*, which is a key vector for many important human arboviruses. As a result, various species, such as *Tx. splendens*, *Tx. rutilus*, and *Tx. amboinensis*, have been proposed as biological control agents against pest species [2,3,4].

As a consequence of adult *Toxorhynchites* being exclusively nectarivorous, they are not considered to be natural vectors for arboviruses. However, previous work has demonstrated that several species of *Toxorhynchites* are susceptible to important arboviruses and, as such, have a role as artificial hosts for their detection and propagation. *Tx. amboinensis*, *Tx. brevipalpis, Tx. rutilus rutilus*, *Tx. theobaldi*, and *Tx. splendens* have previously been shown to be susceptible to a number of flaviviruses including dengue virus (DENV) serotypes 1–4, Japanese encephalitis virus (JEV), yellow fever virus (YFV), and Zika virus (ZIKV) [5,6,7]. Furthermore, certain species have also demonstrated the capacity for generating greater viral titers, especially each of the four DENV serotypes, compared to vector species or mammalian cells commonly used to produce virus. For instance, *Tx. amboinensis* and *Tx. brevipalpis* generate greater titers of DENV compared to *Ae. albopictus* or their derived cell line, C6/36 [5,8]. *Tx. amboinensis* were also shown to be susceptible to JEV and allowed it to replicate to high titres [5]. In addition to DENV and other flaviviruses, *Tx. amboinensis* have been shown to efficiently propagate alphaviruses (chikungunya (CHIKV), Ross River (RRV), and Venezuelan equine encephalitis (VEEV) viruses) and bunyaviruses (La Crosse (LACV), San Angelo (SAV), and Keystone (KEYV)) viruses [5,9,10].

Several continuous cell lines have been derived from *Toxorhynchites* to facilitate virus propagation and isolation in vitro. Cell cultures derived from *Tx. amboinensis* have been established which show comparative levels of sensitivity as the adults and commonly used vector cell lines to DENV and other arboviruses [11,12,13,14]. These cultures provide a useful in vitro system for the study of interactions between arboviruses and *Toxorhynchites* mosquitoes.

Despite their usability for the propagation of arboviruses, nothing is known about the antiviral responses in this mosquito genus. In nature, *Toxorhynchites* spp. may become exposed to arboviruses by predating on vertically infected larvae [15], and it is therefore valuable to understand their antiviral capabilities when considering their use as an alternative to chemical pesticides against vector species. Historically, much of our understanding of mosquito immunity came from extensive research carried out in the *Drosophila melanogaster* model, although an increasingly detailed picture of mosquito immunity in vector species is now emerging which highlights a number of key differences [16,17,18,19,20,21]. The major antiviral mechanism for the control of arboviral infections in mosquitoes is RNA interference (RNAi), which is divided into several pathways differentiated by their effector proteins, small RNA molecules, and their method of induction. The exogenous small interfering RNA (exo-siRNA), and to a lesser extent, the PIWI-interacting RNA (piRNA) pathways are highly important in the context of a viral infection [22,23,24,25,26,27,28,29,30,31,32,33,34,35,36,37,38,39]. The exo-siRNA pathway detects the production of virus-derived long double-stranded RNA (dsRNA). These dsRNAs are cleaved into 21 nucleotide (nt) long virus-specific siRNAs (vsiRNAs) by the exoribonuclease, Dicer 2 (Dcr2). The vsiRNAs are transferred to the RNA-induced silencing complex (RISC) and loaded into the effector protein, Argonaute 2 (Ago2). While one strand of the vsiRNA duplex is degraded, Ago2 uses the other strand to recognize complementary viral RNA, which leads to the cleavage and degradation of the target sequence. The piRNA pathway is not as well-characterized and it’s antiviral role(s) are less clear [40]. It also differs considerably in mosquitoes compared to *D. melanogaster* [41]. In *D. melanogaster*, the pathway involves PIWI proteins Piwi, Aub, and Ago3. However, *Ae. aegypti* lack orthologues of Aub and Piwi, but express Ago3 and an additional 7 PIWI family proteins, Piwi1-7 [41]. The pathway involves piRNA molecules, which are between 24–29 nt in length and are generated through a “ping-pong” amplification system. Intermediate piRNAs are initially produced against genomic transposons and display a characteristic uridine as the first nucleotide (U_1_). These are loaded into the Piwi complex and are further processed to produce mature piRNAs with an adenine at the 10th nucleotide position (A_10_). The mature piRNAs are bound by Ago3 and target complementary antisense RNA transcripts to produce more piRNAs. Therefore, a typical characteristic of ping-pong derived piRNAs is not only the A_10_ and U_1_ bias but also a high frequency of 10 nt complementarity to opposing small RNAs.

In this study, we describe an active antiviral immune response in *Tx. amboinensis*-derived TRA-171 cells. Our observations indicate that these cells possess a functional RNAi response that is effective against Semliki Forest virus (SFV, *Togaviridae, Alphavirus*) infection. We used deep sequencing analysis to show the production of both vsiRNAs and virus-specific piRNAs derived from SFV. In addition, silencing assays showed that RNAi responses are induced by the presence of sequence-specific dsRNA against both viral RNAs and mRNAs transcribed from transfected plasmid DNA. This evidence suggests that *Tx. amboinensis* is able to mount a classical RNAi immune response against viral infections in a similar manner to what is known for mosquito vector species.

## 2. Materials and Methods

### 2.1. Cell Lines

*Tx. amboinensis*-derived TRA-171 cells (European Collection of Authenticated Cell Cultures (ECACC), 90120514) were grown in media prepared in house consisting of L-15 (Leibovitz) growth culture medium (Life Technologies, Carlsbad, CA, USA) mixed 1:1 with Mitsuhashi and Maramorosch basal media prepared in house (CaCl_2_2H_2_O (250 mg/lt), MgCl_2_6H_2_O (125 mg/lt), KCl (250 mg/lt), NaHCO_3_ (150 mg/lt), NaCl (8750 mg/lt), NaH_2_PO_4_H_2_O (250 mg/lt), d-glucose (500 mg/lt), lactalbumin hydrolysate (8125 mg/lt), and yeastolate (0.75 mL/lt)) supplemented with 10% tryptose phosphate broth (TPB, Life Technologies), 10% fetal bovine serum (FBS, Life Technologies), 0.05% bovine serum albumin (BSA) (Sigma-Aldrich, St. Louis, MO, USA), 1% non-essential amino acids (Sigma-Aldrich), and penicillin-streptomycin (final concentration 100 units/mL, 100 µg/mL respectively, Life Technologies). *D. melanogaster*-derived S2 cells [38,42] were cultured in Schneider’s growth media supplemented with 10% FBS and penicillin-streptomycin (final concentration 100 units/mL, 100 µg/mL, respectively). TRA-171 and S2 cells were maintained at 28 °C with no additional CO_2_. Baby hamster kidney (BHK-21) [43] cells were grown in Glasgow’s minimal essential medium (GMEM, Life Technologies) supplemented with 10% TPB, 10% newborn calf serum (NBCS, Life Technologies), and penicillin-streptomycin (final concentration 100 units/mL, 100 µg/mL, respectively) at 37 °C with 5% CO_2_.

### 2.2. Viruses

The prototype molecular clone of SFV, SFV4, and two derived reporter viruses either expressing Firefly luciferase (*FFLuc*) (SFV4(3H)-*FFLuc*) or *Renilla* luciferase (*RLuc*) (SFV4(3H)-*RLuc*) inserted between duplicated nsP2 cleavage sites at the nsP3/4 junction, were grown and titered by plaque assay in BHK-21 cells, as described previously [24]. Infections were performed for 1 h at 28 °C by diluting virus stocks in the appropriate volume of PBSA (phosphate buffered saline with 0.75% BSA) before removing the inoculum and applying fresh growth media.

### 2.3. Plasmids

The *FFLuc* and *RLuc* luciferase expression plasmids, pIZ-Fluc and pAcIE1-Rluc, have been previously described [37,38,44,45].

### 2.4. In Vitro Transcription of dsRNA

dsRNA molecules against either *FFLuc* or *RLuc* were produced using a T7 RNA polymerase in vitro transcription kit (Megascript RNAi kit, Ambion, Foster City, CA, USA) using a PCR product template flanked by T7 RNA polymerase promoter sequences. pIZ-Fluc [45] and pRL-CMV (Promega, Madison, WI, USA) were used as a template for the amplification of dsRNAs targeting *FFLuc* and *RLuc*, respectively. An eGFP-derived dsRNA was taken as a control. This sequence was obtained from a gel-purified PCR product using peGFP-C1 (Clontech, Mountain View, CA, USA) as a template. Primer sequences can be found in Table S1.

Internally radio-labelled dsRNAs were prepared by combining 5 µL 114 nt eGFP PCR product with T7 polymerase sites, 4 µL 5× Transcription buffer (Ambion), 2 µL DTT (0.1 M, Invitrogen, Carlsbad, CA, USA), 1 µL rNTPs (10 mM each ATP, GTP and UTP with 0.1 mM CTP) (Promega), 3 µL α-^32^P rCTP (Perkin Elmer, Waltham, MA, USA), 1 µL T7 RNA polymerase (Ambion), 1 µL RNase inhibitor (Invitrogen), and 3 µL RNase/DNase free H_2_O. The reaction was incubated for 1 to 3 h at 37 °C before heating to 65 °C for 5 min and allowing it to gradually cool to room temperature. Following this, 2 µL of DNase I and 1 µL of RNase A were added and the reaction was incubated for a further 30 min at 37 °C. The dsRNA could then be purified by running on an 8% native acrylamide gel.

### 2.5. Nucleic Acid Transfection

Cells were seeded at a density of 2.2 × 10^5^ cells per well of a 24-well plate 24 h prior to transfection with Dharmafect 2 (GE Healthcare, Chicago, IL, USA) following the manufacturer’s instructions. For plasmid transfection experiments, each well was co-transfected with 100 ng pIZ-Fluc, 100 ng pAcIE1-Rluc (as an internal control) with either 1 ng of dsRNA (*FFLuc*-specific or eGFP control dsRNA), or 2 ng siRNAs (*FFLuc*-specific or Hygromycin B resistance gene control siRNA [39]). Cells were lysed 24 h post transfection (p.t.) and luciferase activity determined.

For infection experiments, viral reporter gene transcripts were silenced by transfecting 50 ng *RLuc* or eGFP-specific control dsRNA. After 24 h, cells were infected with SFV4(3H)-*RLuc* at a multiplicity of infection (MOI) of 0.005. Cells were lysed 24 h post infection (p.i.) and luciferase activities were determined.

### 2.6. Luciferase Assay

The cells were lysed in Passive Lysis Buffer (Promega) and luciferase expression was determined with either the Renilla-Glo Luciferase assay system (Promega) or the Dual Luciferase assay system (Promega) and a GloMax luminometer.

### 2.7. Small RNA Sequencing and Analysis

The cells were grown at a density of 9 × 10^5^ per well of a six-well plate. RNA extraction was performed using 1 mL TRIzol (Life Technologies) as per the manufacturer’s instructions, with the addition of glycogen as a carrier. DNA libraries consisting of small RNAs between 15–40 nt were gel purified and sequenced using the Illumina Hiseq 4000 platform at BGI Tech (Shenzhen, China). Data analysis was carried out as described previously by aligning sequence reads to the SFV4 reference genome (Genbank accession number: KP699763) [24,37]. A maximum of one mismatch or indel was allowed in the alignments. Alignment lengths between 18–36 nt were selected for further analysis. These were separated into two groups according to their orientation, i.e., if they mapped to the genome (positive) or antigenome (negative). Coverage plots of mapped reads were generated using R programming language. Sequence logos were generated using a Bioconductor package, motifStack [46]. The mapping positions of each small RNA that aligned to the positive strand of the SFV4 genome was compared to the positions of small RNAs that aligned to the negative strand (antigenome). Any overlaps between these were recorded. Similarly, small RNA pairs of 25–29 nt were compared and their overlapping nucleotide frequencies were aggregated [47]. Using python program coding and the R statistical package, standard scores (z-scores) of the overlapping nucleotides and their frequencies were calculated and plotted. Small RNA sequencing data is available at Sequence Read Archive (https://www.ncbi.nlm.nih.gov/sra) under the accession number: PRJNA486770.

### 2.8. In Vitro Dicer Cleavage Assay

TRA-171 and S2 cells were seeded at 9 × 10^5^ and 1 × 10^6^ cells per well of a six-well plate respectively. Following a 24 h incubation, the media was removed. The cells were re-suspended in sterile PBS and centrifuged for 5 min at 1500 rpm. The supernatant was removed and the procedure repeated a second time as described. Following this, the pellet was re-suspended in 200 µL 1× lysis buffer (10 mM MgAc (Sigma-Aldrich) and 150 mM Hepes-KOH (pH 7.5)) and homogenized using a micro-pestle. A further centrifugation step (14,000 rpm for 20 min at 4 °C) was performed to remove cell debris, after which 5 µL of the supernatant was transferred to a fresh tube. To this was added, 3 µL ^32^P labelled dsRNA, 1 µL H_2_O, and a 3 µL creatine mix (consisting of 1 µL DTT (1 M), 10 µL creatine phosphate (12 mg/100 µL) (Calbiochem, San Diego, CA, USA), 20 µL 5× lysis buffer (10 mM MgAc and 150 mM Hepes-KOH (pH 7.5)), 20 µL glycerol, 2 µL RNase Inhibitor (Promega), 2 µL ATP (100 mM) (Thermo Fisher Scientific, Waltham, MA, USA), 0.3 µL 20 mg/mL creatine phosphate kinase in 1× storage buffer (40 mg/mL lyophilized creatine kinase (Calbiochem) in 2× storage buffer (ice-cold 40 mM tris-acetate (pH 6.8), 200 mM KAc, 0.2 mM EDTA, 20 mM β-mercaptoethanol) diluted 1:1 in an equal volume of 100% ice-cold glycerol), and 4.7 µL RNase/DNase free H_2_O). The reactions were incubated at 28 °C overnight. Subsequently, 200 µL 2× PK buffer (200 mM Tris (pH 7.5), 300 mM NaCl, 5 mM EDTA, 2% SDS), 1 µL glycogen (10 mg/mL) (Roche, Basel, Switzerland), and 0.3 µL proteinase K (10 mg/mL) (Sigma-Aldrich) were added and each reaction was incubated for a further 10 min at 65 °C. Following this, 200 µL phenol/chloroform/isoamylalcohol (25:24:1) (Ambion) was added. Reactions were vortexed for 15 sec and centrifuged for 10 min at 10,000 rpm. The aqueous phase was transferred to a new tube containing 450 µL ice-cold 96% EtOH and centrifuged at 13,000 rpm for 10 min. The supernatant was removed and the pellet washed with 400 µL ice cold 70% EtOH. The samples were further centrifuged as described and the supernatant was removed. The resulting pellets were air dried for 5–10 min prior to resuspension in 15 µL 2× RNA gel loading buffer (Thermo Fisher Scientific). The samples were boiled for 5 min at 65 °C before placing on ice for 2 min. On completion, the samples were loaded onto a 0.75 mm 12% denaturing acrylamide gel with 0.96% urea chilled by submerging the tank in ice-cold water. Electrophoresis was carried out at 200 V. The gel was then transferred to a gel dryer and allowed to dry at 80 °C for 2 h. The resulting bands were detected by exposing the gel to a phosphor imaging screen for ≥16 h and viewed using a personal molecular imager (Bio-Rad, Hercules, CA, USA).

### 2.9. Statistical Analysis

Statistical analysis was performed using GraphPad Prism. Data was analyzed using an unpaired, two-tailed *t*-test.

### 2.10. Data Availability

Source data for the figures can be found at http://dx.doi.org/10.5525/gla.researchdata.703. Small RNA sequencing data can be found under the accession number described above.

## 3. Results

### 3.1. SFV Infects Tx. amboinensis-Derived TRA-171 Cells

**Figure 1:**
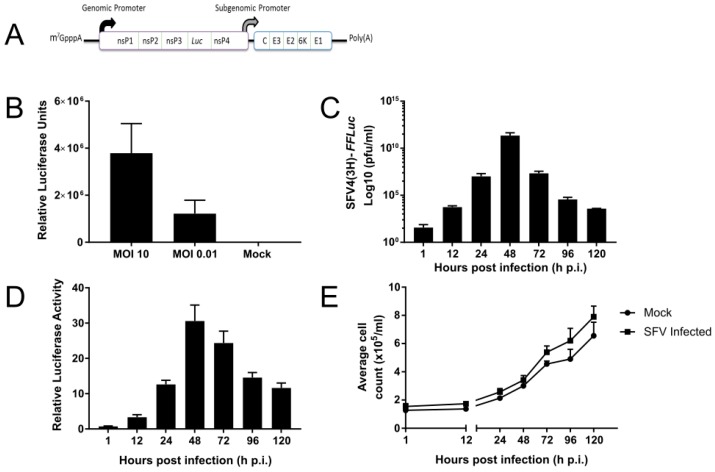
Infection of TRA-171 cells with SFV. (**A**) Schematic representation of the design of the reporter strains of SFV expressing luciferase (*Luc*: either *RLuc*, SFV4(3H)-*RLuc*, or *FFLuc*, SFV4(3H)-*FFLuc*) inserted between duplicated nsP2-protease cleavage sites at the nsP3/4 junction. (**B**) TRA-171 cells were either mock infected or infected with SFV4(3H)-*RLuc* at MOI 10 or 0.01. Luciferase expression was determined at 24 h p.i. by luciferase assay and relative luciferase activity was normalized against background, shown on the *Y*-axis. (**C**) TRA-171 cells were infected with SFV4(3H)-*FFLuc* at MOI 10. Cell growth media was collected and replaced with fresh media at the given time points. Virus production was determined by plaque assay and the titre was measured in plaque forming units (PFU/mL). (**D**) The cells were infected as in (**C**) and lysed at the given time points to monitor viral replication. Luciferase expression was determined by luciferase assay and relative luciferase units are shown on the *Y*-axis. (**E**) The cells were either infected as in (**C**) (■) or mock infected (●) and cell numbers were counted at the given time points. Mean values with standard error are shown for three (**B**) or two (**C**–**E**) independent experiments conducted in triplicate. The analysis used the average of each triplicate per experiment.

*Tx. amboinensis*-derived TRA-171 cells are known to be permissive to infection by CHIKV [14], but it has not been shown that they can be infected by SFV, a related alphavirus. To answer this question, TRA-171 cells were infected with SFV expressing luciferase (either *RLuc* or *FFLuc*) (Figure 1A), which allows replication to be monitored directly. Cells were infected with SFV4(3H)-*RLuc* at a high (10) or low (0.01) MOI and incubated for 24 h prior to lysing. As anticipated, proportional SFV replication was detected at each MOI used (Figure 1B). To understand virus kinetics during infection, the cells were infected with a second SFV reporter strain (SFV4(3H)-*FFLuc*) at MOI 10 and its replication efficiency was monitored at regular intervals over 120 h. Both viral titres (Figure 1C) and luciferase expression (Figure 1D) peaked after 48 h p.i. before decreasing. It was also determined that cell numbers between infected and uninfected cultures were similar and increased at a comparable rate over the 120 h observation period (Figure 1E). These observations are consistent with previous studies that show a similar pattern of SFV infection in *Ae. albopictus*-derived cell lines [48,49]. This data therefore suggests that SFV infection in the TRA-171 cell line displays similar kinetics to an arboviral infection in vector cells.

### 3.2. Functional RNAi Pathways are Present in TRA-171 Cells

**Figure 2:**
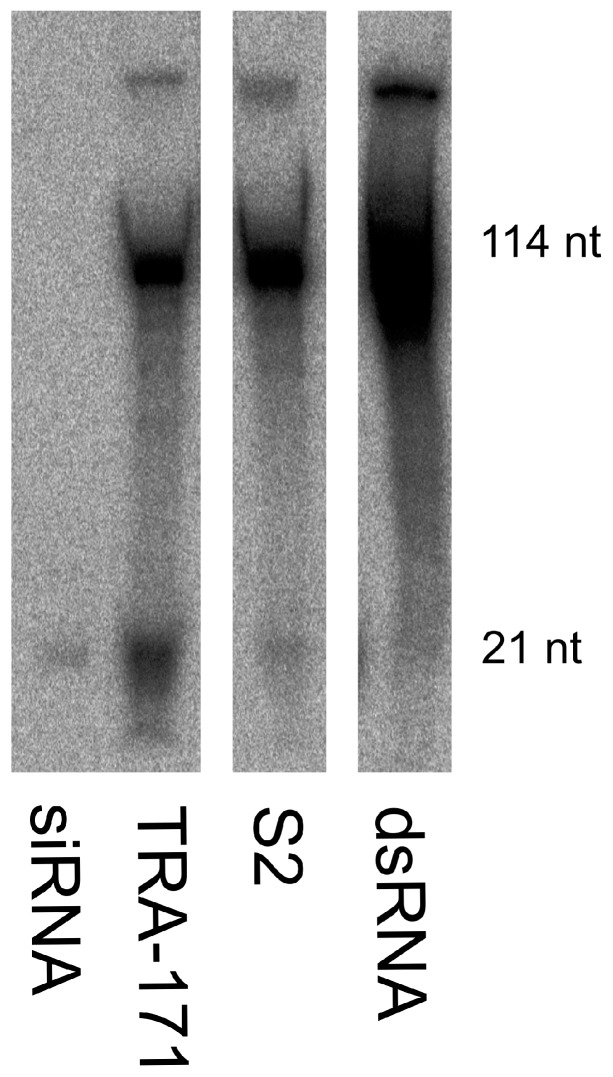
Production of small RNAs from long dsRNA. The cellular extracts were prepared from TRA-171 and S2 cells. The extracts were incubated with ^32^P internally-labeled dsRNA (114 nt). Size markers of long dsRNA (114 nt) and siRNAs (21 nt) are indicated to show approximate sizes. The image shown is representative of three independent experiments and shows relevant individual lanes. The complete image is shown in Supplementary Figure S1.

A distinguishing feature of the exo-siRNA pathway in vector mosquitoes is that, through Dcr2 cleavage of dsRNA, it produces 21 nt siRNAs that are complementary to the target sequence. To investigate if TRA-171 cells express a functional Dicer enzyme and are thereby capable of generating siRNAs, we used an in vitro Dicer cleavage assay. The cell extracts were incubated with ^32^P internally radio-labeled dsRNA and incubated overnight before isolating the small RNAs. Extracts were run on an acrylamide gel alongside size markers; input dsRNA (114 nt) and siRNAs (21 nt), as well as an extract from S2 cells as a positive control. Both samples showed the detection of input dsRNA in addition to discernible bands at the size expected for 21 nt siRNAs (Figure 2, Figure S1). This data suggests that TRA-171 cells possess an active dicing enzyme that is effectively able to cleave long dsRNA molecules into small RNAs of approximately the expected size for siRNAs.

**Figure 3:**
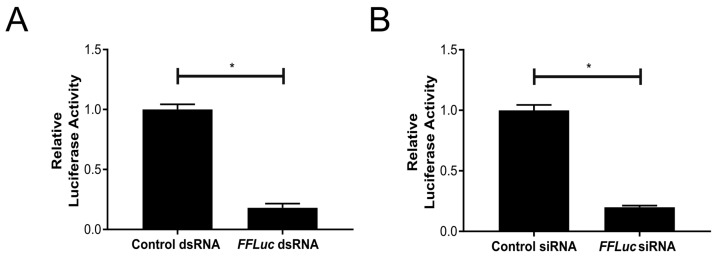
dsRNA or siRNA mediated gene silencing in TRA-171 cells. The cells were co-transfected with pIZ-Fluc and pAcIE1-Rluc (as an internal control) alongside either dsRNA (**A**) or siRNAs (**B**) targeting *FFLuc* or a control. Luciferase expression was determined by luciferase assay 24 h p.t. and relative luciferase activity (*FFLuc*/*RLuc*) is shown on the *Y*-axis. Mean values with standard error are shown for four independent experiments performed in triplicate. The analysis used the average of each triplicate per experiment. * indicates significance, *p* < 0.05 by Student *t*-test.

The siRNA pathway is induced in a sequence specific manner through the detection of dsRNAs by Dcr2. To determine the ability of TRA-171 cells to silence a *FFLuc* reporter gene via the RNAi pathway, we performed a previously described reporter RNAi assay [50]. Cells were co-transfected with both pIZ-Fluc and pAcIE1-Rluc (as an internal control), as well as either *FFLuc*-specific or negative control dsRNA/siRNAs. Each condition was lysed 24 h p.t. and luciferase expression assessed. Our data demonstrates that the cells that received either *FFLuc*-specific dsRNAs (Figure 3A) or siRNAs (Figure 3B) showed a significant decrease in relative luciferase activity compared to the cells treated with control dsRNA/siRNAs. This suggests that TRA-171 cells are able to induce a gene silencing response, which is mediated by the presence of both sequence-specific dsRNA and siRNAs.

### 3.3. An Active dsRNA-Inducible RNAi Response has Antiviral Activity against SFV Infection in TRA-171 Cells

**Figure 4:**
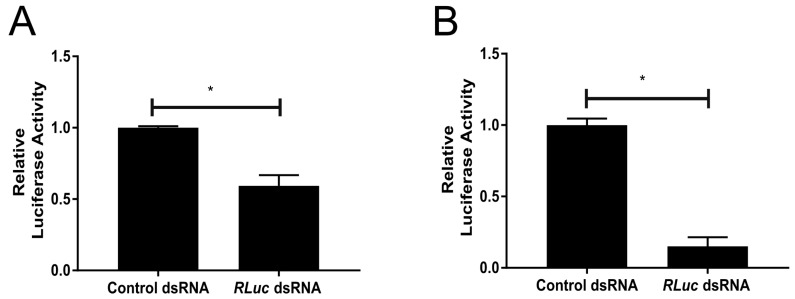
TRA-171 cells possess a dsRNA-inducible antiviral RNAi pathway. The cells were transfected with dsRNA against either *RLuc* or eGFP (as a control) 24 h prior to being either mock infected or infected with SFV(3H)-*RLuc* at MOI 10 (**A**) or 0.005 (**B**). Luciferase expression was determined by luciferase assay 24 h p.i. and luciferase activity is shown on the *Y*-axis. Mean values with standard error are shown for three independent experiments performed in triplicate. The analysis used the average of each triplicate per experiment. * indicates significance, *p* < 0.05 by Student *t*-test.

Next, we assessed if this silencing pathway has an inducible antiviral function against SFV. The cells were first treated with either *RLuc*-specific or eGFP-specific control dsRNA prior to infection with SFV4(3H)-*RLuc*. The infections were performed at either a high (10) or low (0.005) MOI. The cells were lysed 24 h p.i. and luciferase expression was assessed. A decrease in the relative luciferase activity was observed in cells treated with *RLuc*-specific dsRNA when compared to those that received control eGFP-specific dsRNA at both MOIs, which directly indicates a decrease in viral replication and, therefore, a reduction in virus production (Figure 4A,B). These findings suggest that TRA-171 cells possess a sequence specific antiviral response that can be externally induced by dsRNA.

### 3.4. SFV Infection Induces Small RNA Production in TRA-171 Cells

**Figure 5:**
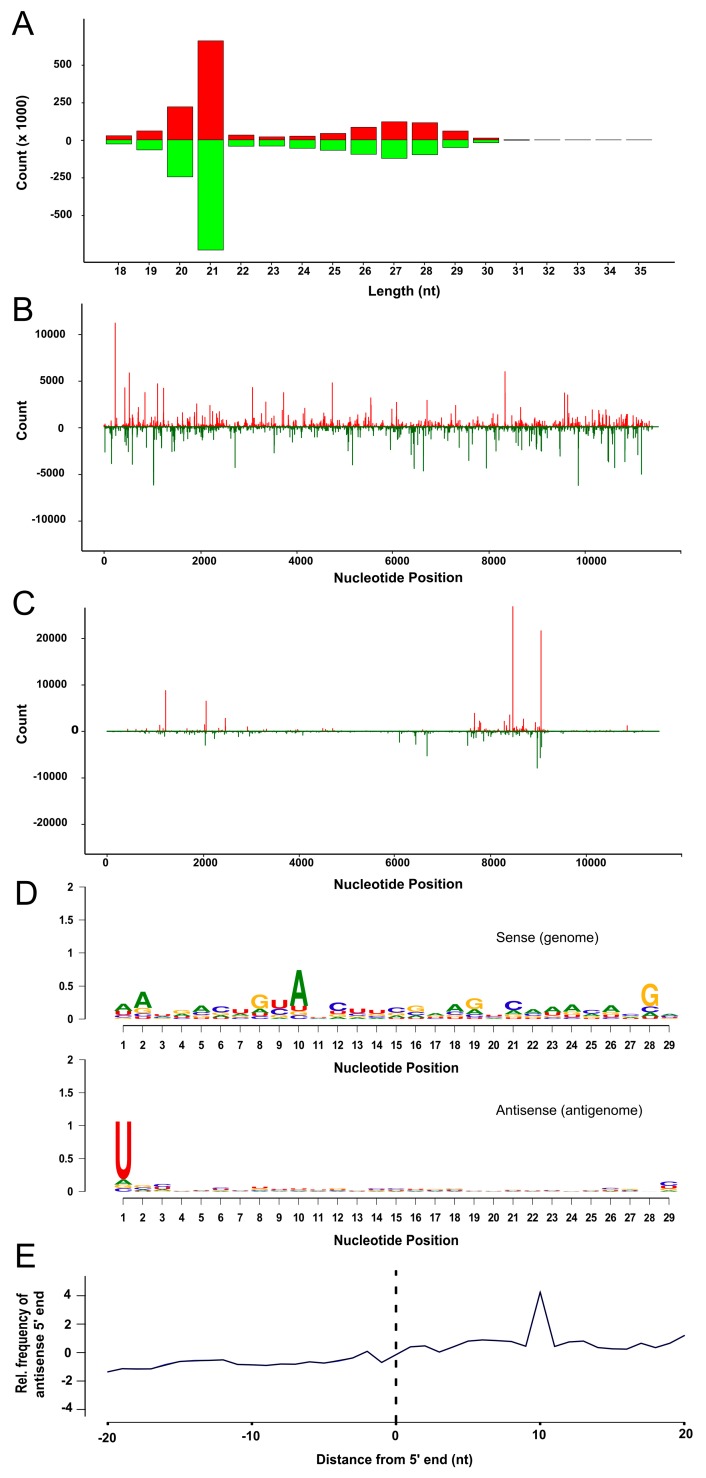
Characteristics of SFV-derived small RNAs in TRA-171 cells. RNA was isolated from TRA-171 cells 24 h p.i. with SFV4 at MOI 10 followed by small RNA sequencing. (**A**) The size distribution of small RNAs from SFV infected cells mapping to the SFV4 genome (red, positive numbers) or antigenome (green, negative numbers). The distribution of 21 nt (**B**) or 28 nt (**C**) small RNAs across the length of the SFV genome (red, positive numbers) or antigenome (green, negative numbers). The *Y*-axis shows the frequency of small RNAs mapping to the corresponding nucleotide location on the *X*-axis. (**D**) The conserved relative nucleotide frequency at each position of 29 nt long small RNAs mapping to the SFV4 genome or antigenome represented on a web logo diagram. The height of each nucleotide represents the degree of sequence conservation. The level of conservation is indicated by the *Y*-axis. (**E**) Frequency map showing the distance between the 5′ ends of 25–29 nt small RNAs mapping to the opposite strand of the SFV4 reference sequence. Position 0 represents the first nucleotide. The results shown are representative of two independent experiments.

It has previously been shown that, following infection, non-vector mosquito cells are capable of generating small RNAs of the expected size and properties expected of vsiRNAs and vpiRNAs [22,50]. Having shown that TRA-171 cells have an inducible RNAi response that is capable of controlling SFV replication, we next wanted to establish if TRA-171 cells have the capacity to generate vsiRNAs and/or vpiRNAs which specifically target SFV. TRA-171 cells were infected with SFV4 at MOI 10 and RNA isolated 24 h p.i. for sequencing. Small RNAs were sequenced and analyzed by mapping to both the SFV genome and the antigenome (Figure 5, Supplementary Table S2). SFV-specific vsiRNAs predominantly 21 nt in length were found to be produced within infected cells (Figure 5A). These reads were found to map to both the viral genome and the antigenome in approximately equal quantities. The pattern of vsiRNA distribution observed (Figure 5B) indicates regions of many reads (hot spots) and regions with few reads (cold spots). A second class of SFV-specific small RNAs were also identified, which ranged from 24–29 nt and again mapped to both the genome and antigenome of SFV (Figure 5A). These preferentially targeted specific regions of the coding strand (Figure 5C) and presented with the characteristic signature of piRNAs; a bias for A at position 10 and a U at position 1 (Figure 5D). The 5′ ends of these complementary small RNAs overlapped most frequently by 10 nt, which suggests that these are vpiRNAs produced via the ‘ping-pong’ mechanism (Figure 5E). Taken together, this data suggests that the antiviral RNAi response in TRA-171 cells is induced following SFV infection and produces both vsiRNAs and vpiRNAs which target viral sequences.

Other analysis of the sequencing data revealed the presence of putative novel insect specific viruses (ISVs), although further studies are needed to confirm their presence (Supplemental Materials and Methods, Supplemental Table S3 and Figure S2). ISVs lack the ability to replicate in vertebrate cells but have been naturally shown to infect a variety of arthropods in nature including mosquitoes and are present in several mosquito derived cell lines [51,52,53,54,55,56,57,58,59]. It will be beneficial to assess their impact on the host and their involvement in pathogen transmission in order to understand how this may affect vector competence and arbovirus transmission within specific populations. How these ISVs, as well as other sequences such as transposable elements (TEs) and endogenous viral elements (EVEs), interact with the RNAi response will be important to develop a more complete awareness of the global role of RNAi out with arboviral infections. For instance, given the known correlation between EVEs and the piRNA pathway [60], this information may provide relevant data for understanding how viruses establish a persistent infection and the potential for mosquitoes to pass on heritable immune indicators.

## 4. Discussion

Studies in mosquito vector species have shown that RNAi is the predominant antiviral response against infections [17,19,20]. Specifically, the exo-siRNA has been demonstrated to be important in regulating this antiviral activity, although the antiviral role of piRNAs is unclear. Previous research has used *D. melanogaster* as a model for RNAi studies [61,62,63,64,65,66,67,68,69], but very few studies have investigated the role of the RNAi pathways in non-vector mosquito species. *Toxorhynchites* is one of three mosquito genera, along with *Malaya* and *Topomyia*, which do not require a blood meal during their adult life stages to initiate egg development and, as such, do not exhibit host-seeking behavior [70,71]. Their lack of importance as a medically relevant pest species has meant that their general biology has been largely neglected. However, in their role as a biological control agent against pest species, *Toxorhynchites* may be at risk of an arbovirus infection due to ingesting vertically infected larvae [15]. Therefore, it is important to understand their antiviral capabilities.

This study identifies the presence of a functional RNAi response within *Tx. amboinensis*-derived TRA-171 cells. SFV infection induced the production of 21 nt vsiRNAs derived from both the viral genome and antigenome, which is indicative of Dcr2 cleavage of dsRNA [20]. A similar enrichment of 21 nt vsiRNAs has previously been reported for SFV, as well as other alphaviruses [24,26,28,31,32,37,39,72] and members of the *Bunyaviridae* and *Flaviviridae* [18,19,20,21]. Deep sequencing of SFV-infected TRA-171 cells indicates that these vsiRNAs are distributed across the genome and antigenome with hot spot and cold spot areas. This is consistent with previous SFV data obtained from aedine cell lines [24,28,37,39]. Further work would be required to determine whether vsiRNAs derived from cold spot regions are able to inhibit SFV replication significantly more effectively than hot spot derived vsiRNAs, as has been shown previously [28].

In addition, larger classes of SFV-derived small RNAs between 24–29 nt were detected which presented with the hallmark characteristic ping-pong motif of piRNAs; an A_10_/U_1_ bias and a 10 nt overlap between the 5′ ends of piRNAs from different strands, which has been previously described in aedine cells [24,31,32,37]. Consistent with previous findings for SFV infection of Aag2 and U4.4 cells, these were less widely distributed and preferentially targeted specific regions of the coding strand [24,31,32]. However, unlike data from aedine cells, these vpiRNAs are present in approximately equal quantities against both sense and antisense sequences, rather than with a bias towards the sense strand. The identification of 24–29 nt small RNAs displaying a A_10_ and U_1_ bias and a 10 nt overlap between the 5′ ends of different strands suggests that TRA-171 cells encode PIWI clade proteins.

Our findings show that the exo-siRNA pathway can be artificially induced in TRA-171 cells by the transfection of long dsRNA molecules, which leads to sequence-specific silencing. Similarly, our results also show that sequence-specific siRNAs are capable of achieving efficient gene silencing, which supports the presence of a natural antiviral RNAi pathway within TRA-171 cells. Key mediator proteins of the exo-siRNA pathway, such as Dcr2 and Ago2, are highly conserved between mosquito and drosophila and the effector mechanisms are considered to be similar [73]. Previous studies have confirmed the importance of these proteins as mediators of infection as viral replication increases following their knockdown [23,24,27,33,37,74]. Similarly, recent studies in *Ae. aegypti* show that Ago3, Piwi5, and, to a lesser extent, Piwi6 participate in the production of viral-derived piRNAs [35,36]. Given the lack of genomic information available for *Tx. amboinensis*, it was not possible to identify RNAi effector-encoding sequences and, therefore, we can only speculate if these proteins are present in the *Toxorhychites* genome. However, the results presented here are a strong indicator for the presence of RNAi machinery comparable to that of aedine species. Previous research has shown that arboviruses are able to replicate to high levels within *Toxorhynchites* mosquitoes and their derived cell lines. The data shown here for SFV replication and production is congruous with these findings. Further work will be required to determine the exact temporal and spatial mechanisms, as well as the proteins involved in the *Toxorhynchites* immune response, which permit enhanced arbovirus replication.

In conclusion, we have demonstrated the presence of an active antiviral RNAi response in *Toxorhynchites* cells which successfully modulates SFV infection in a manner similar to that of natural arboviral vector species. Only once we fully understand their ability to manage infections will we be able to make an informed decision regarding the suitability of certain species as biological control agents against vector mosquito species. Our data also expands on the current knowledge of ISVs, TEs, and EVEs in mosquitoes that may influence pathogen transmission in vector populations and highlights their interactions with the exo-siRNA and piRNA pathways.

## Supplementary Materials

The following are available online at http://www.mdpi.com/1999-4915/10/12/694/s1, Supplemental Materials and Methods: Small RNA analysis for the identification of novel putative insect specific viruses, Figure S1: Production of small RNAs from long dsRNA, Figure S2: Characteristics of contigs derived from an EVE, TE, and a potential novel virus, Table S1: Primer sequences, Table S2: Sequencing reads results, Table S3: Overview of assembled contigs showing similarity to viruses or TEs.
