# Supplementary material for: Antiviral RNA Interference Activity in Cells of the Predatory Mosquito, Toxorhynchites amboinensis
Source: Viruses. 2018 Dec 6;10(12):694. doi: 10.3390/v10120694 (PMC6316411; doi:10.3390/v10120694)
Supplement: Supplementary file 1 [file viruses-10-00694-s001.pdf]

<u>Supplemental Material</u>

Supplemental Materials and Methods:

Small RNA analysis for the identification of novel putative insect specific viruses

Virome analysis based on small RNA libraries was conducted as described previously [1]. Briefly, small RNA libraries were pre-processed in order to filter out reads below the minimum quality (phred 20) or those containing ambiguous nucleotides and for the removal of sequencing adaptors. Small RNAs mapping to the genome reference of SFV4 were also removed. Remaining reads greater than 15 nt were used to assemble contigs using Velvet assembler [2] with a fixed k-mer of 15 and an automatically determined k-mer defined by the Velvet optimiser script. Consolidation of contigs derived from different assemblies was performed using Cap3 [3]. Contiguous sequences larger than 50 nt were characterised through sequence similarity searches against GenBank databases followed by analysis of the size profile of small RNAs. Endogenous and exogenous viral sequences were determined using molecular characteristics of small RNAs mapping to each substrate, as described previously [4]. Briefly, contigs that showed sequence similarity to viral sequences and had small RNA profiles consistent with production of siRNAs were classified as derived from an exogenous virus, while those that showed small RNA profiles consistent with piRNAs were classified as derived from endogenous viral elements (EVEs). Small RNA profiles were assessed by comparing 15-35 nt reads to each reference sequence using Bowtie software [5] and allowing one mismatch. Size distribution, density of coverage, sequence logos and distance between the 5′ end of piRNAs in different strands were calculated and plotted using in-home Perl and R programming languages using ggplot2 [6] and motifStack [7] packages.

SFV-derived contigs were discarded from the posterior analysis and the remaining library examined for its similarity to viral or transposable element (TE) sequences at the protein level. Although sequence similarity searches indicated the presence of viral sequences in each of the four libraries, library N-204 (SFV4-infected) was chosen for further processing as it presented the highest number of assembled contigs and reads from other viral sequences (Supplementary Table S2). Some of these contigs showed similarity to known viral references at the protein level and were further analysed (Supplementary Table S3). These viral sequences could be separated into potential viruses or endogenous viral elements (EVEs) derived from non-reverse transcribing RNA viruses known to be present in the genome of many animals. An example of a putative new virus and an EVE (related to Whidbey virus) identified in our analysis were used to create small RNA profiles, along with data mapping to a putative TE sequence related to a gypsy element found in *Tabanus bromius* (Supplementary Table S3, Supplementary Figure S2). New viruses were distinguished based on the presence of both sequence-specific siRNAs and piRNAs. These were distributed across the genome and antigenome, with hot and cold spots regions; however, unlike the SFV-derived piRNAs, the putative virus piRNAs did not show the classic ‘ping-pong’ signature. EVE and TE hits presented with a small RNA profile that only matched piRNAs, specifically those that mapped to the sense strand. These piRNAs did show the expected U_1 bias.

Supplemental Results

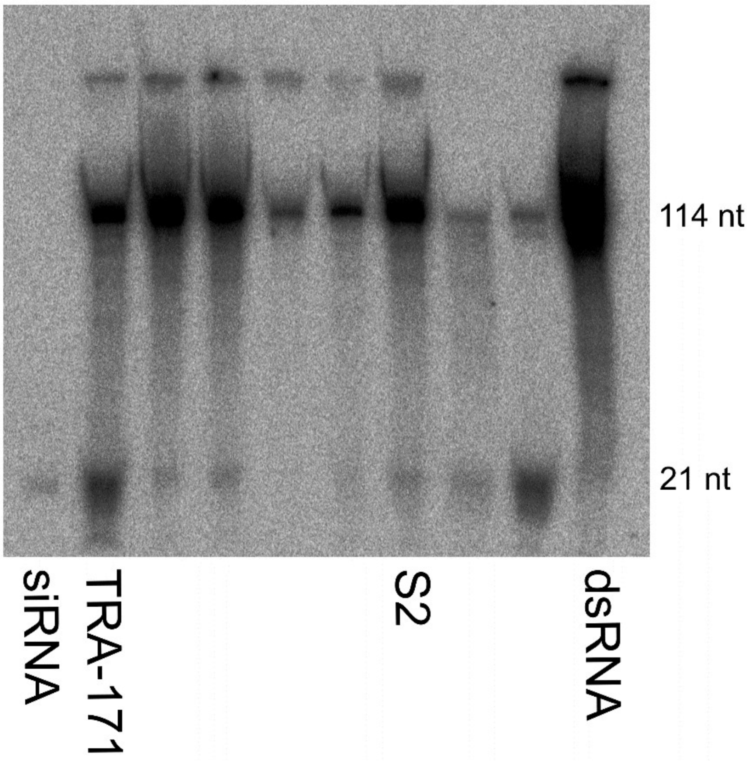

Figure S1. Full image of Figure 2A. Relevant lanes shown in Figure 2A are labelled.

Table S1. Primer sequences**.** All sequences are shown 5′-3′. Underlined bases indicates the T7 promoter sequence.

Primer	Sequence	Use
RLuc FWD	TAATACGACTCACTATAGGGATGACTTCGAAAGTTTATGATCCAG	dsRNA
RLuc RV	TAATACGACTCACTATAGGGCTGCAAATTCTTCTGGTTCTAACTTTC	
FFLuc FWD	GTAATACGACTCACTATAGGGACTTACGCTGAGTACTTC	dsRNA
FFLuc RV	GTAATACGACTCACTATAGGGGAAATCCCTGGTAATCCG	
eGFP 400 FWD	GTAATACGACTCACTATAGGGGGCGTGCAGTGCTTCAGCCGC	dsRNA
eGFP 400 RV	GTAATACGACTCACTATAGGGGTGGTTGTCGGGCAGCAGCAC	
eGFP 114 FWD	GTAATACGACTCACTATAGGGGGCGTGCAGTGCTTCAGCCGC	^{32}P labelled dsRNA
eGFP 114 RV	GTAATACGACTCACTATAGGGGCCGTCCTTGAAGAAGATGG	

Table S2. **An overview of the number of reads obtained from each of the four libraries.**

Library	Infection	# total reads	# after processing*	#assembled contigs◊	#viral contigs	#reads SFV	#reads from other viral sequences
N-202	SFV	46,439,835	13,405,454	300	94	2,311,379	169,365
N-203	Mock	54,094,620	12,921,898	263	43	7,624	220,030
N-204	SFV	53,733,782	13,857,134	346	87	2,112,339	228,092
N-205	Mock	52,709,867	12,563,539	283	32	6,472	217,359

*Reads were filtered based on quality, ambiguous bases and size. ◊Assembled contigs are greater than 50 nt**.**

Table S3. Overview of assembled contigs in library N-204 showing similarity at the protein level to viruses or transposable elements.

Contig ID	Size (nt)	E-value	Closest sequence in Genbank	Classification based on the small RNA profile
Contig2197_2196_455	186	3,00E-07	putative glycoprotein [Gambie virus]	EVE
Contig72_71	103	7,00E-04	hypothetical protein 4 [Hubei lepidoptera virus 5]	New virus
Contig572_571_465	136	4,00E-20	putative glycoprotein [Imjin River virus 1]gb|ALP32029.1	EVE
Contig2220_2219_460	183	7,00E-08	NP [Whidbey virus]	EVE
Contig200_199	216	1,00E-19	NP [Whidbey virus]	EVE
Contig236_235_456	120	8,00E-09	putative glycoprotein [Gambie virus]	EVE
**Contig198_197**	**216**	**2,00E-05**	**NP [Whidbey virus]**	**EVE**
Contig6_454	130	6,00E-11	putative RNA-dependent RNA polymerase-like protein, partial [uncultured virus]	New virus
Contig197_196	251	1,00E-11	putative RNA-dependent RNA polymerase-like protein, partial [uncultured virus]	New virus
Contig201_200	125	5,00E-04	Retrovirus-related polyprotein of transposon [Anoplophora glabripennis]	TE
**Contig793_792_457**	**121**	**2,00E-09**	**putative gypsy retrovirus-related pol polyprotein,**	**TE**

			**partial [*Tabanus bromius*]**	
**Contig1_451_199_198**	**352**	**4,00E-53**	**putative RNA-dependent RNA polymerase-like protein [uncultured virus]**	**New virus**

In **bold**, contigs that were used to plot the molecular signature of small RNAs in Figure 6.

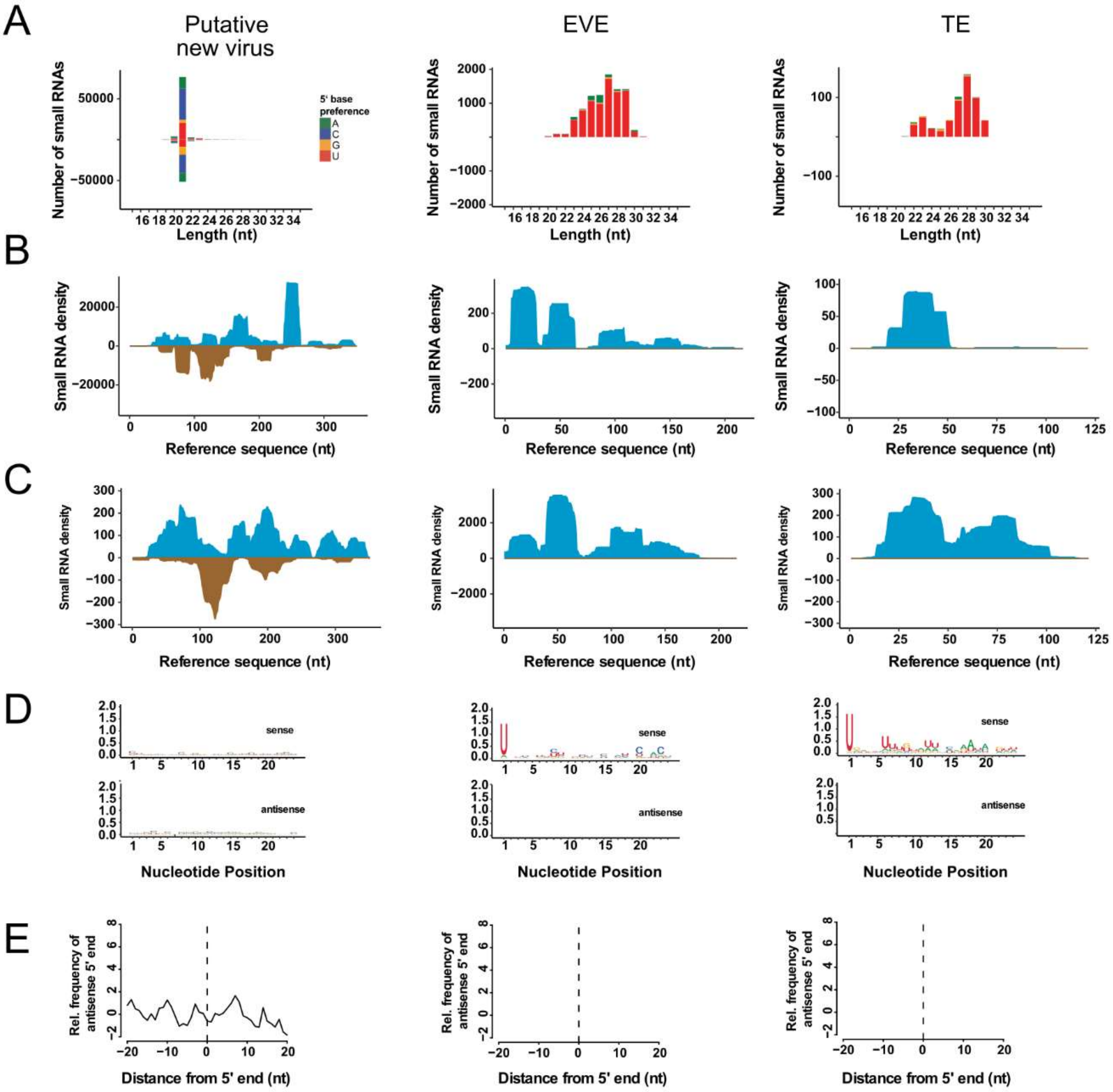

Figure S2. Characteristics of contigs derived from an EVE, TE and a potential virus found in TRA-171 cells. **(A)** Size distribution of small RNAs from TRA-171 cells which map to the sequence of a possible new virus, an EVE or a TE (Table 1). The different bases are represented by different colours. The density of 20-23 nt **(B)** or 24-29 nt **(C)** small RNAs distributed across the sense (blue, positive numbers) or antisense (brown, negative numbers) strands of the reference sequences. **(D)** Relative nucleotide frequency at each position of small RNAs between 24-29 nt mapping to the sense or antisense orientation of the reference sequence described in Table 1. The level of conservation is indicated on the Y-axis. **(E)** Relative frequency map showing the distance between 5′ ends of 24-29 nt small RNAs mapping to opposite strand of the reference sequence (Supplementary Table S3). Position 0 represents the first nucleotide. The results shown are representative of two independent experiments.

References

1. Aguiar ER, Olmo RP, Paro S, Ferreira FV, de Faria IJ, Todjro YM, et al. Sequence-independent characterization of viruses based on the pattern of viral small RNAs produced by the host. Nucleic Acids Res. 2015;43(13):6191-206. doi: 10.1093/nar/gkv587. PubMed PMID: 26040701; PubMed Central PMCID: PMCPMC4513865.
2. Zerbino DR, Birney E. Velvet: algorithms for de novo short read assembly using de Bruijn graphs. Genome Res. 2008;18(5):821-9. Epub 2008/03/20. doi: 10.1101/gr.074492.107. PubMed PMID: 18349386; PubMed Central PMCID: PMCPMC2336801.
3. Huang X, Madan A. CAP3: A DNA sequence assembly program. Genome Res. 1999;9(9):868-77. Epub 1999/10/06. PubMed PMID: 10508846; PubMed Central PMCID: PMCPMC310812.
4. Aguiar ER, Olmo RP, Marques JT. Virus-derived small RNAs: molecular footprints of host-pathogen interactions. Wiley interdisciplinary reviews RNA. 2016. doi: 10.1002/wrna.1361. PubMed PMID: 27170499.
5. Langmead B. Aligning short sequencing reads with Bowtie. Curr Protoc Bioinformatics. 2010;Chapter 11:Unit 11 7. Epub 2010/12/15. doi: 10.1002/0471250953.bi1107s32. PubMed PMID: 21154709; PubMed Central PMCID: PMCPMC3010897.
6. Wickham H. ggplot2: elegant graphics for data analysis: Springer; 2016.
7. Ou J, Wolfe SA, Brodsky MH, Zhu LJ. motifStack for the analysis of transcription factor binding site evolution. Nat Methods. 2018;15(1):8-9. doi: 10.1038/nmeth.4555. PubMed PMID: 29298290.
